# Frontoamygdala hyperconnectivity predicts affective dysregulation in adolescent moderate-severe TBI

**DOI:** 10.3389/fresc.2022.1064215

**Published:** 2023-01-04

**Authors:** Kevin C. Bickart, Alexander Olsen, Emily L. Dennis, Talin Babikian, Ann N. Hoffman, Aliyah Snyder, Christopher A. Sheridan, Jesse T. Fischer, Christopher C. Giza, Meeryo C. Choe, Robert F. Asarnow

**Affiliations:** ^1^ BrainSPORT, Department of Neurosurgery, UCLA, Los Angeles, CA, United States; ^2^ Department of Neurology, UCLA, Los Angeles, CA, United States; ^3^ Department of Psychiatry and Biobehavioral Sciences, UCLA, Los Angeles, CA, United States; ^4^ Department of Psychology, Norwegian University of Science and Technology, Trondheim, Norway; ^5^ Department of Physical Medicine and Rehabilitation, St. Olavs Hospital, University Hospital, Trondheim, Norway; ^6^ TBI and Concussion Center, Department of Neurology, University of Utah, Salt Lake City, UT, United States; ^7^ Wake Forest School of Medicine, Radiology Informatics and Image Processing Laboratory, Winston-Salem, NC, United States; ^8^ Wake Forest School of Medicine, Department of Radiology, Section of Neuroradiology, Winston-Salem, NC, United States; ^9^ UCLA Mattel Children's Hospital, Department of Pediatrics, Division of Neurology, Los Angeles, CA, United States

**Keywords:** amygdala, resting-state fMRI, moderate to severe TBI, affective dysregulation, frontoamygdala, behavioral dysregulation

## Abstract

In survivors of moderate to severe traumatic brain injury (msTBI), affective disruptions often remain underdetected and undertreated, in part due to poor understanding of the underlying neural mechanisms. We hypothesized that limbic circuits are integral to affective dysregulation in msTBI. To test this, we studied 19 adolescents with msTBI 17 months post-injury (TBI: M age 15.6, 5 females) as well as 44 matched healthy controls (HC: M age 16.4, 21 females). We leveraged two previously identified, large-scale resting-state (rsfMRI) networks of the amygdala to determine whether connectivity strength correlated with affective problems in the adolescents with msTBI. We found that distinct amygdala networks differentially predicted externalizing and internalizing behavioral problems in patients with msTBI. Specifically, patients with the highest medial amygdala connectivity were rated by parents as having greater externalizing behavioral problems measured on the BRIEF and CBCL, but not cognitive problems. The most correlated voxels in that network localize to the rostral anterior cingulate (rACC) and posterior cingulate (PCC) cortices, predicting 48% of the variance in externalizing problems. Alternatively, patients with the highest ventrolateral amygdala connectivity were rated by parents as having greater internalizing behavioral problems measured on the CBCL, but not cognitive problems. The most correlated voxels in that network localize to the ventromedial prefrontal cortex (vmPFC), predicting 57% of the variance in internalizing problems. Both findings were independent of potential confounds including ratings of TBI severity, time since injury, lesion burden based on acute imaging, demographic variables, and other non-amygdalar rsfMRI metrics (e.g., rACC to PCC connectivity), as well as macro- and microstructural measures of limbic circuitry (e.g., amygdala volume and uncinate fasciculus fractional anisotropy). Supporting the clinical significance of these findings, patients with msTBI had significantly greater externalizing problem ratings than healthy control participants and all the brain-behavior findings were specific to the msTBI group in that no similar correlations were found in the healthy control participants. Taken together, frontoamygdala pathways may underlie chronic dysregulation of behavior and mood in patients with msTBI. Future work will focus on neuromodulation techniques to directly affect frontoamygdala pathways with the aim to mitigate such dysregulation problems.

## Introduction

Traumatic brain injury (TBI) continues to be a leading cause of death and disability in youth (1, 2). Estimates suggest that in a single year, pediatric TBI in the United States leads to 630,000 emergency department visits, 60,000 hospitalizations, and 7,500 deaths. Nearly 40% of youth survivors (3), or approximately 145,000 individuals per year (4), suffer from long-term disability. Affective problems after pediatric moderate/severe TBI (msTBI) represent a common, underdetected, and undertreated subset of disability, limiting cognitive, emotional, social, and behavioral functioning. In many cases, these affective problems meet diagnostic thresholds for psychiatric conditions (5) and disrupt normal development, academic performance, and interpersonal relationships (1, 6, 7). Specific symptoms involve externalizing behaviors, such as poor impulse-control and aggression (8–11) and internalizing behaviors, such as depression, anxiety, and avoidance (12–17). Unfortunately, no standard treatment for affective problems exists for TBI, due in part to a lack in understanding the neural mechanisms of such problems.

Normal affective function relies on limbic pathways that may be particularly vulnerable to the contusional and torsional forces of brain trauma, including less granular portions of the prefrontal and temporal cortices as well as their subcortical targets in the medial temporal lobe, striatum, hypothalamus, and brainstem (18–21). TBI-related aberrancies in limbic regions have been found using measures of brain macro- and microstructure (16, 20–27), task-based fMRI (28–30), and resting-state fMRI (31–34). Furthermore, animal studies suggest brain injury has both local effects on the tissue at the site of the trauma, but also indirect effects on the connectional architecture of the brain that are remote from the site of trauma (35). Nevertheless, limbic networks have been studied far less than neocortical networks (31–34), and few studies have combined functional with structural imaging modalities to interrogate the circuitry underlying affective disruption in TBI (31, 34, 36, 37). Prior studies also lack specific hypotheses about the functional neuroanatomy of the limbic brain.

**Figure 1:**
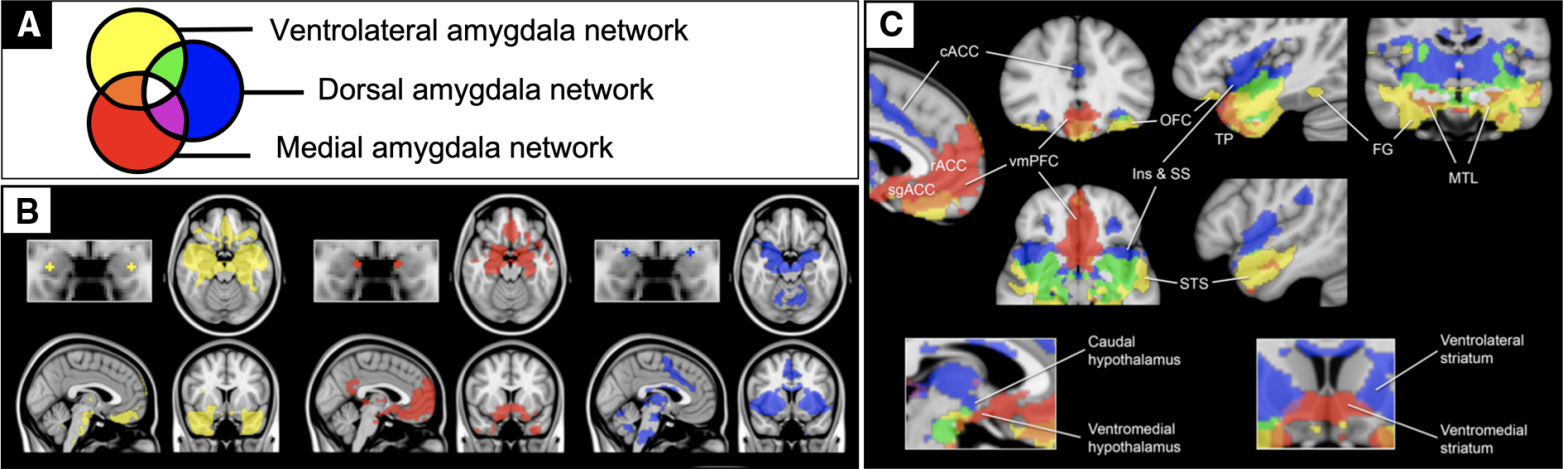
Previously published rsfMRI functional connectivity maps of the amygdala. Previously published one sample group mean significance maps color coded (**A**) for each of 3 amygdala seeds (*N*=89) displayed in standard views (**B**) and selected views for network differentiation (**C**). The maps are binarized at *p* < 10^−5^ and overlaid on a T1 MNI152 0.5 mm template brain in radiologic convention to demonstrate the distinct and shared connectivity across maps (38).

The present study leveraged a systems neuroscience framework for limbic circuitry centered on the amygdala (Figure 1). We previously defined three anatomically distinct amygdala networks using resting-state fMRI connectivity (rFC) in healthy individuals (38). These networks support distinct aspects of affective behavior (38, 39), serve as endophenotypes for genetic drivers of such behaviors (40), and degenerate in relation to distinct affective symptoms in frontotemporal dementia (41). This framework includes a network supporting the perception of salient stimuli containing sensory association regions (yellow), a network supporting the regulation of approach behavior and autonomic activity containing goal- and reward-related regions (red), and a network supporting avoidance learning and behavior containing threat- and pain-responsive regions (blue).

Although the resting-state correlates of affective and behavioral dysregulation are understudied in adolescent TBI, prior work in other populations provide some direction for hypotheses. Specifically, studies on typical development (42) and conduct disorder (43, 44) converge to suggest that elevated connectivity between the amygdala and rostral anterior cingulate portends more externalizing problems. Given this connection is unique to the medial amygdala (red in Figure 1), we hypothesized here that patients with more externalizing behaviors would have increased medial amygdala network connectivity. Internalizing behaviors also seem to be more prevalent in people with altered amygdala connectivity (45–47), but particularly with a more ventral region within the ventromedial prefrontal cortex (48). Given this connection is unique to the ventrolateral amygdala network (yellow in Figure 1), we hypothesized here that patients with more internalizing behaviors would have increased ventrolateral amygdala network connectivity. We then tested whether these relationships were specific to functional connectivity measures over and above contributions from demographic, injury, and structural imaging measures.

## Materials and methods

### Participants

We studied patients after a non-penetrating msTBI who were recruited from four different pediatric intensive care units in Los Angeles County. Inclusion criteria included a Glasgow Coma Scale (GCS) score between 3 and 12 (or a GCS above 12 with abnormalities on clinical imaging), aged between 8 and 18, normal or corrected-to-normal visual acuity, and proficient English competence to understand instructions for the neuropsychological inventories. Exclusion criteria included prior TBI, neurologic deficits that would preclude them from completing any of the planned testing, or prior neurologic, developmental, or serious psychiatric diagnoses. For comparison, 46 age- and sex-matched healthy controls (HC) were recruited through flyers, magazines, and school postings. Controls met the same inclusion and exclusion criteria as the TBI group other than those pertaining to the brain injury under investigation. All patients who underwent MRI were also required to be eligible for scanning (e.g., no metal implants or shrapnel). The institutional review boards of University of California, Los Angeles (UCLA) and the other sites of recruitment approved this study.

The study protocol has been previously described in detail (49). In the present study, we report the first analysis focused on rsfMRI data from this protocol. Of the 50 patients with TBI recruited, 19 had rsfMRI scans from the chronic time frame (TBI: 13–22 months). Clinical and demographic details are presented in Table 1.

**Table 1:** Demographic and clinical variables for TBI and HC groups.

	TBI	HC	*p-*value: 2
N: Total (Female)	19 (5)	44 (21)	0.11
Age: M (SD) years	15.6 (2.7)	16.4 (2.6)	0.28
Hand: R/l	16/3	40/4	0.45
Mean framewise displacement: M (SD)	0.1 (0.8)	0.1 (0.1)	0.69
Percentage of volumes removed: M (SD)	2.2 (3.8)	1.6 (3.3)	0.54
GCS on arrival: M (SD)	9.3 (3.6)	–	–
Time since injury: M (SD) weeks	67.7 (9.0)	–	–
Intracranial pressure issues: #	1	–	–
Diffuse axonal injury: #	0	–	–
Subarachnoid hemorrhage: #	4	–	–
Ventricular hemorrhage: #	2	–	–
Epidural hematoma: #	6	–	–
Subdural hematoma: #	4	–	–
Intracerebral hematoma: #	8	–	–
Contusion: #	5	–	–
Non-depressed skull fracture: #	4	–	–
Depressed skull fracture: #	7	–	–
Motor vehicle accident: #	11	–	–
Fall: #	6	–	–
Blunt trauma: #	2	–	–

*The last column displays resulting *p-*values from the independent samples *t*-tests of demographic variables across TBI > HC, respectively. TBI traumatic brain injury; HC healthy control group; N sample size; M mean; SD standard deviation; GCS Glasgow Coma Scale.

### Image acquisition

All patients underwent MRI scans on a 3T Siemens Trio with a 12-channel Head Matrix Coil (Siemens AG). Patients were instructed to remain still throughout the scan and foam pads were placed around their heads to further minimize head motion. For rsfMRI, T2* weighted BOLD fMRI was acquired while Patients rested with their eyes open for 7 min and 55 s, utilizing an echo-planar imaging (EPI) pulse sequence (TR = 2400 ms, TE = 35 ms, FOV = 244 mm, matrix = 80 × 80, slice thickness = 3 mm, number of slices = 40, and flip angle = 90° giving an in-plane resolution of 3 × 3 mm). For anatomical reference, T1-weighted 3D MPRAGE images were acquired (TR = 1900 ms, TE = 3.26 ms, FOV = 250 mm, slice thickness = 1 mm, matrix = 256 × 256, and flip angle = 9°, giving an in-plane resolution of 1 × 1 mm). For microstructural metrics, diffusion weighted imaging (DWI) was acquired (GRAPPA mode; acceleration factor PE 2; TR 9500 ms; TE 87 ms; FOV 256 × 256 mm; isotropic voxel size 2 mm; 72 images per subject with 8 b0 and 64 diffusion-weighted, b 1,000 s/mm^2^).

### Resting-state fMRI processing and analysis

Processing and analysis of the MRI data were performed using the CONN Toolbox version 19b (50) and Statistical Parametric Mapping version 12 (SPM12) in Matlab 2018b. Scans were preprocessed using the default pipeline, which includes realignment, slice-timing correction, outlier identification, co-registration, segmentation, normalization and smoothing (8 mm Gaussian kernel). Functional outlier detection was performed *via* Artifact Removal Tools (ART) in CONN using intermediate settings (97th percentile in normative sample). After scrubbing removed volumes with framewise displacement (FD) > 0.9 mm, we removed 2 subjects from the HC group from further analyses because their scrubbed time series had an overall mean FD > 0.9 mm. All rsfMRI and T1 images were visualized for lesions that would overlap with the networks of interest; no lesions or encephalomalacia were observed in close vicinity to the amygdala network masks used. Scrubbed time series were then passed into CONN's denoising steps, including band-pass filtering (0.008–0.09 Hz) and regression of noise components generated from individually segmented white matter and cerebrospinal fluid masks (5 components for each *via* CompCor) as well as motion parameters and their first-order derivatives (generated from ART). From the denoised times series for each subject, we computed rFC in the *a priori* defined amygdala networks (Figure 1) and performed second-level analyses on these networks, as described in detail below.

### DTI processing and analysis

DTI data were processed as described previously (51–53). Briefly, we used a tract clustering and identification method developed in our laboratory, automated multi-atlas tract extraction (autoMATE). Raw DTI images were visually checked for artifacts. DTI volumes were corrected for eddy current-induced distortions using the FSL tool *eddy_correct* (http://fsl.fmrib.ox.ac.uk/fsl/) and skull stripped using *BET*. Fractional anisotropy (FA) and mean diffusivity (MD) maps were computed using *dtifit*. Whole-brain HARDI tractography was performed with Camino (http://cmic.cs.ucl.ac.uk/camino/). The maximum fiber turning angle was set to 35°/voxel to limit biologically implausible results, and tracing stopped when FA dropped <0.2, as is the standard in the field. The Eve atlas was registered, linearly and then non-linearly, to each patient's FA map using Advanced Normalization Tools (ANTs) and its ROIs were correspondingly warped to extract 19 tracts of interest, of which we focused on bilateral uncinate fasciculus given it connects the frontal lobe to medial temporal lobe. Five white matter tract atlases were created from healthy controls, and each patient's FA map was linearly and nonlinearly registered to each of these in order to refine fiber extractions, the results of these five tract identifications were fused for each patient for a final, cleaned fiber clustering. Registrations at each step were visually checked for quality. MD was averaged within ROIs to generate summary measures for the medial and ventrolateral amygdala subregions, using the cluster masks previously published (38).

### Morphometric processing and analysis

We used an automated segmentation and probabilistic region-of-interest (ROI) labeling technique (FreeSurfer, http://surfer.nmr.mgh.harvard.edu) to perform a quantitative morphometric analysis of the T1 MRI scans (54). Forty different brain regions are segmented and assigned neuroanatomic ROI labels using probabilistic estimations based on a manually labeled atlas dataset of 40 individuals. All ROIs were divided by the total intracranial volume to control for differing head size, as performed previously (55).

### Behavioral ratings

The Behavior Rating Inventory of Executive Function (BRIEF) (56) is a parent rating of “real world” behavioral manifestations of executive functioning problems. The Child Behavior Checklist (CBCL) is a 112-item parent rating scale targeting various domains of behavioral functioning (57). The current study used standardized subscales of the BRIEF and CBCL in which higher T scores indicate greater problems. On both inventories, parents rated their child's current functioning. At the first assessment, parents were also asked to retrospectively rate their child's pre-injury behaviors in the 6 months predating the TBI.

We were most interested in investigating associations between resting-state connectivity and ratings of mood and behavior regulation. As such, our primary behavioral outcome measures were Emotional Control and Externalizing subscales of the BRIEF and CBCL, respectively. We also assessed the Working Memory subscale of the BRIEF to test whether amygdala network connectivity was specific to mood and behavior regulation rather than more general to frontal regulation phenomena.

### Statistical analysis

All variables of interest were assessed for normality and outliers, removing values greater than 3 standard deviations from the mean. We first compared TBI and HC groups on potential confounding variables using independent samples *t*-tests using SPSS, accepting alpha of 5% as significant.

For whole network analysis, we performed a series of independent first order Pearson correlational analyses in the TBI group to discern whether connectivity for the medial and ventrolateral amygdala networks predicted externalizing and internalizing problems, respectively and specifically, as hypothesized. For the externalizing behavior domain, we used two distinct measures to test for convergent validity, the Emotional Control subscale on the BRIEF and the Externalizing subscale on the CBCL. For the internalizing behavior domain, we did not have a similar measure to test for convergent validity. For both externalizing and internalizing domains, we also examined the discriminant validity of the brain-behavior findings by testing whether amygdala network connectivity correlated with a less affective domain, the Working Memory subscale of the BRIEF. We also tested the specificity of the amygdala network-behavior associations by assessing whether macro- and microstructural metrics within limbic regions and circuits, including uncinate fasciculus FA, medial amygdala MD, and amygdala volume, accounted for any of the predicted variance in the behavior domain of interest. Finally, we tested whether potential confounds accounted for any of the predicted variance, including age, sex, handedness, time since injury, and severity of injury (GCS on arrival), presence of gross injuries on hospital CT (as described in Table 1). We performed each of these tests in SPSS and considered them as independent, accepting alpha of 5% as significant.

Subsequently, we performed voxelwise analyses within the network of interest to determine voxels of greatest correlation with the behavioral measure. We performed these analyses in the CONN Toolbox (50), using the following parameters for multiple comparison correction, *p*-uncorrected ≤ 0.01 with cluster-size *p*-FDR corrected ≤ 0.05. Given that the voxelwise map of the association between medial amygdala connectivity and Emotional Control problem ratings included anterior and posterior cingulate regions that are considered part of the default mode network, we also tested whether connectivity between the anterior and posterior cingulate ROIs from the Brainnetome Atlas (58) (A32sg and A23d, respectively) correlated with Emotional Control problem ratings using the CONN Toolbox ROI to ROI regression analysis, accepting alpha of 5% as significant. Finally, we extracted Fisher r-to-z values for peak voxels from the voxelwise analyses to compare TBI and HC groups using independent samples *t*-tests in SPSS, accepting alpha of 5% as significant.

## Results

### Group differences in demographic and clinical variables

Patients in the TBI groups incurred injuries through a variety of mechanisms, as detailed in Table 1. From the computed tomography (CT) scan that patients received at the hospital prior to study enrollment, the types of neuropathology were detailed for each patient. The TBI group showed no significant differences from the HC group on potential confounding variables (Table 1).

### Externalizing problems: whole network analysis

**Figure 2:**
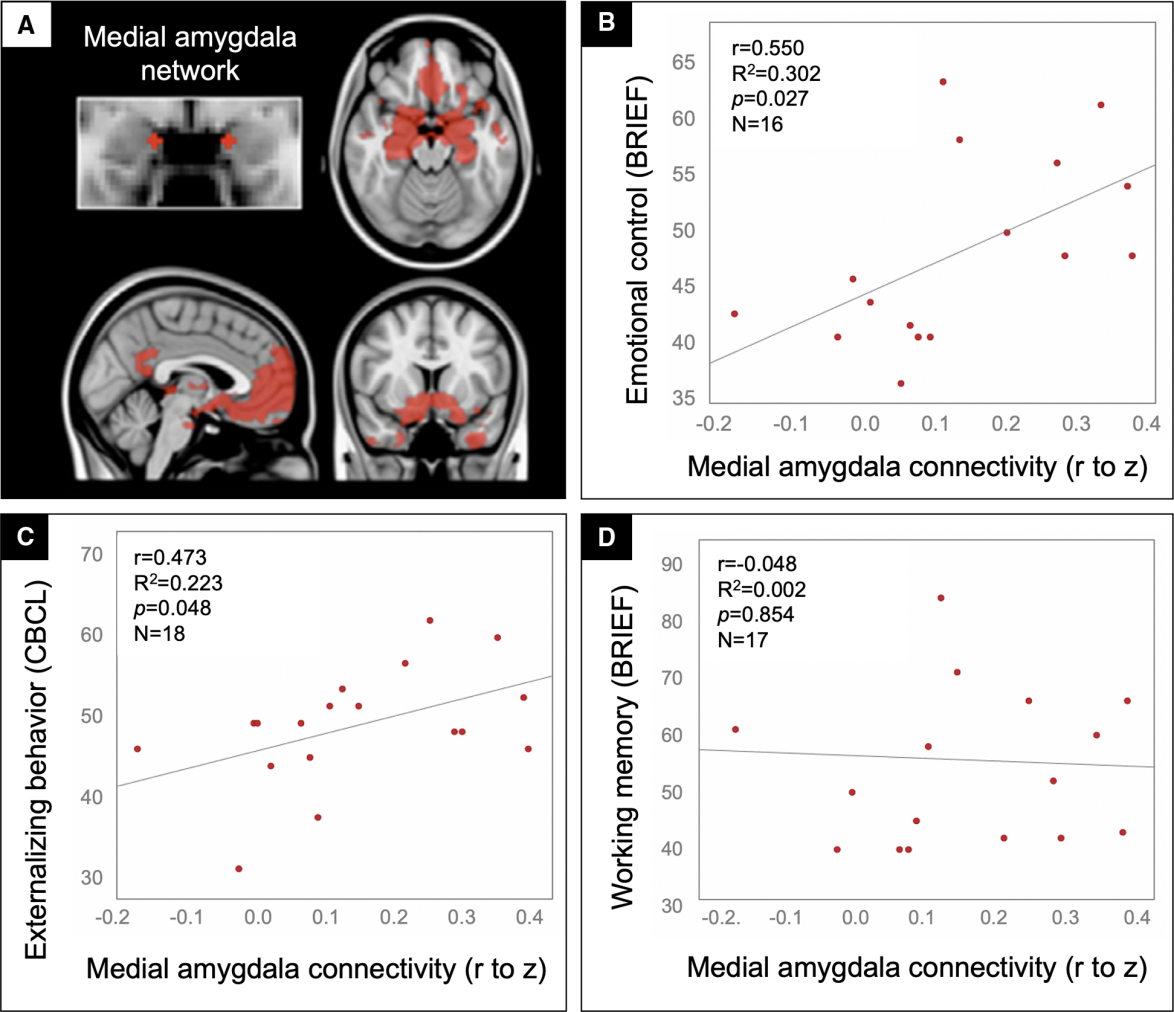
Medial amygdala hyperconnectivity predicted behavioral dysregulation but not working memory problems for patients in the TBI group. Scatter plots showing the correlation between medial amygdala (**A**) rFC values (x-axis) and the BRIEF Emotional control (**B**), CBCL Externalizing behavior (**C**), and BRIEF Working memory (**D**) scale ratings (y-axis) with statistics for the Pearson correlation overlaid.

Given the function of regions in the medial amygdala network (Figure 2A) – regulating affective responses to meet the goals and demands of a situation – and prior work in adolescents implicating regions within this circuit in externalizing behaviors (42, 43), we tested whether connectivity in this network correlated with the Emotional Control subscale of the BRIEF. Patients with the greatest medial amygdala connectivity were rated by their parents to have the most problems with emotional control (Figure 2B). For convergent validity, we examined an independent scale tapping a similar domain, Externalizing Behavior from the CBCL, and again found that patients in the TBI group with the highest connectivity had the highest ratings of externalizing problems (Figure 2C). None of these findings could be explained by potential confounds, including age, sex, handedness, time since injury, severity of injury (GCS on arrival), presence of gross injuries on hospital CT (as described in Table 1), uncinate fasciculus FA, medial amygdala MD, or amygdala volume, in that these variables did not correlate with both amygdala connectivity and the behavioral variables. Further, supporting the specificity of these brain-behavior relationships to the affective domain, we found no significant correlation between medial amygdala connectivity and ratings on the Working Memory subscale of the BRIEF (Figure 2D).

### Externalizing problems: voxelwise analysis

**Figure 3:**
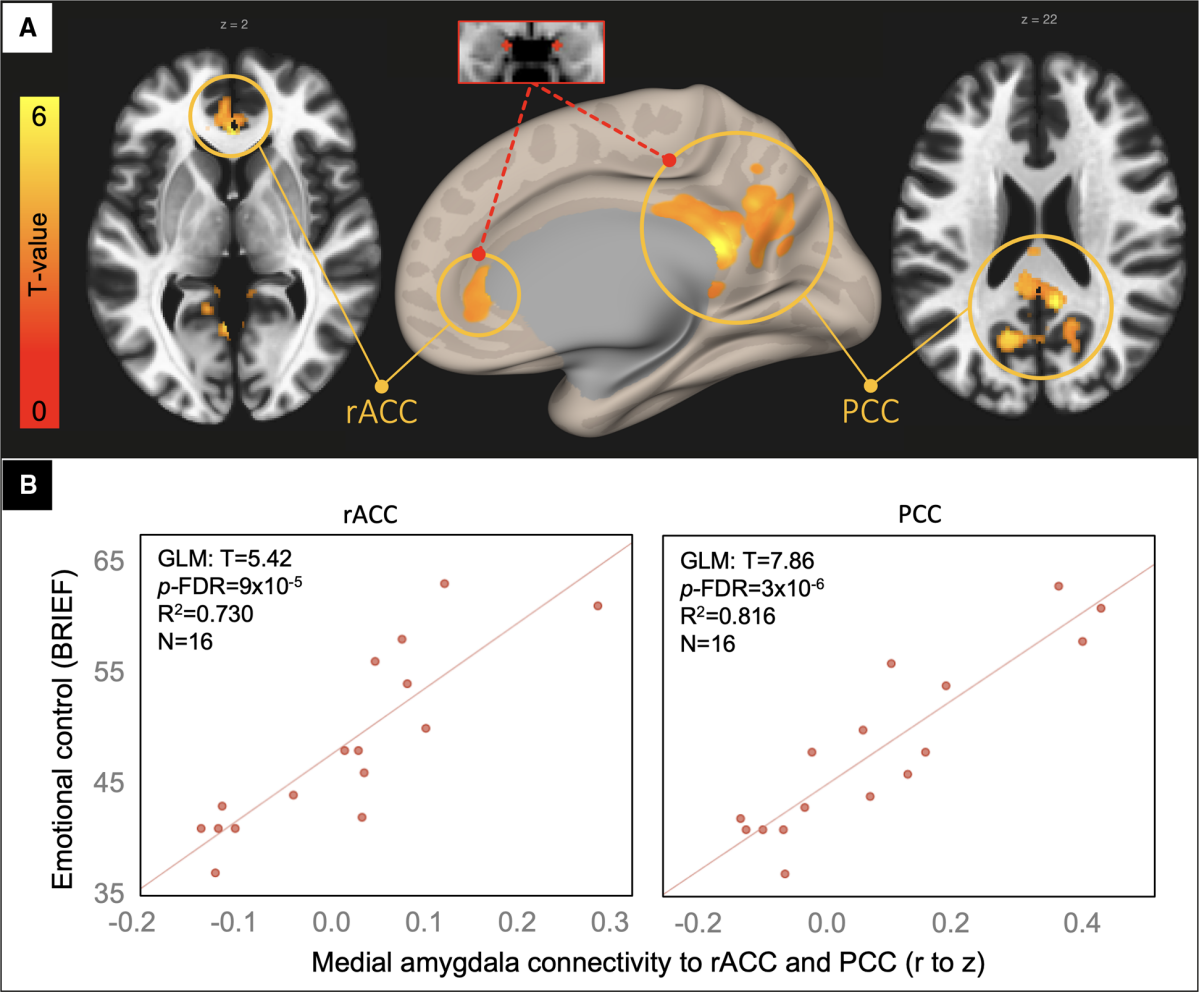
Voxelwise correlation between BRIEF emotional control ratings and medial amygdala connectivity. Resultant clusters of voxels in the rostral anterior cingulate cortex (rACC) and posterior cingulate cortices (PCC) for the regression of BRIEF Emotional Control ratings on medial amygdala rFC (**A**, *p*-uncorrected < 0.01 with cluster-size *p*-FDR corrected < 0.05). Scatter plots **(B)** showing the correlation between rFC of the medial amygdala to peak voxels in the rACC and PCC clusters (x-axis) and the BRIEF scale (y-axis) with overlaid statistics for the GLM on the first row of plots derived from the voxelwise regression.

In a voxelwise analysis of the medial amygdala network, patients in the TBI group with the highest emotional control problem ratings had the greatest connectivity between the medial amygdala and voxels in the rostral anterior and posterior cingulate cortices (rACC and PCC, Figure 3).

Given that the resultant clusters from the voxelwise analysis are part of the canonical default mode network, we investigated whether their connectivity to one another – a good proxy for default mode connectivity – correlated with these behavioral ratings. Again, supporting the specificity of the main finding, we found no significant correlation between rACC to PCC connectivity and the Emotional Control or Externalizing Behavior scales (*p *= 0.33 and 0.59, respectively).

### Internalizing problems: whole network analysis

**Figure 4:**
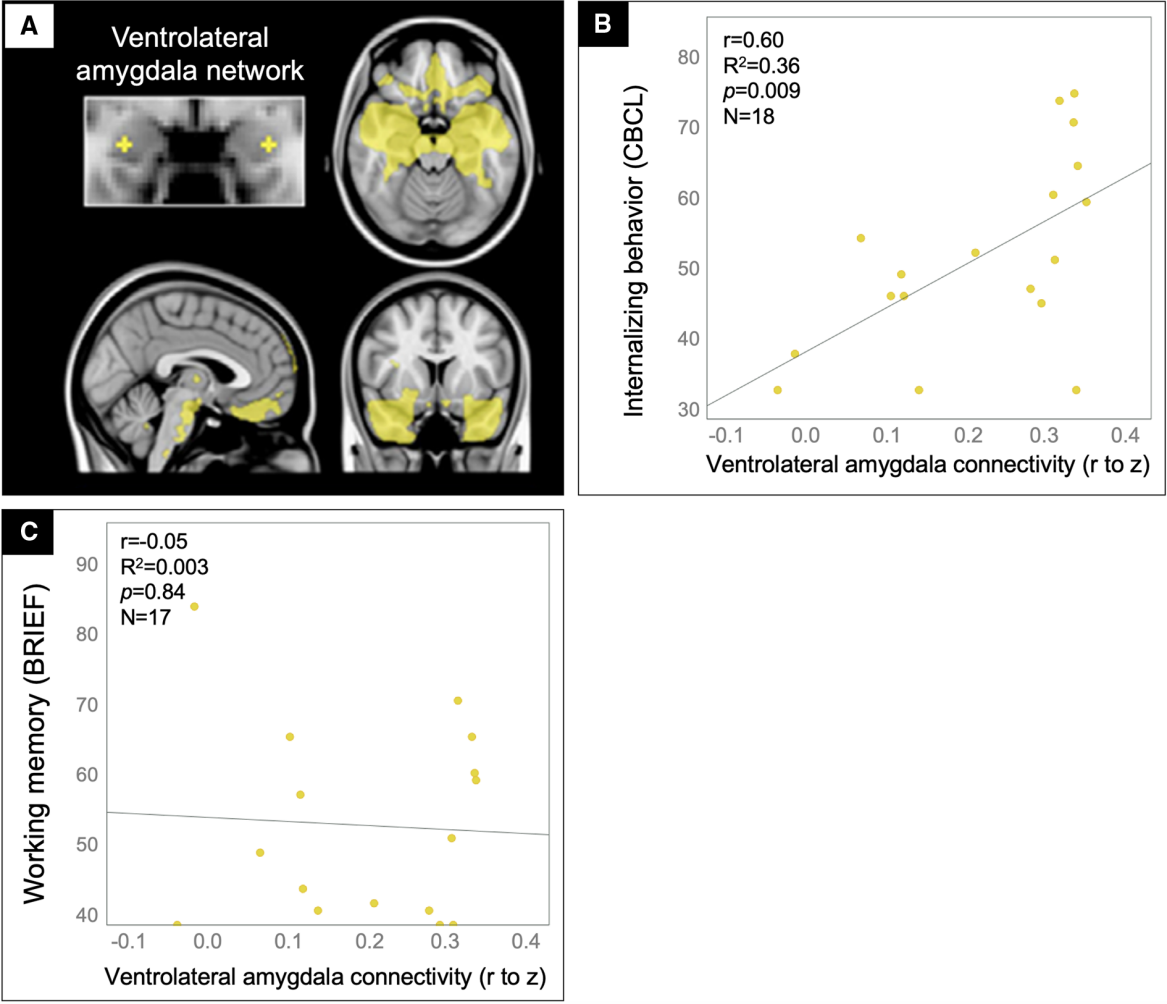
Ventrolateral amygdala hyperconnectivity predicted internalizing problems but not working memory problems for patients in the TBI group. Scatter plots showing the correlation between ventrolateral amygdala (**A**) rFC values (x-axis) and the CBCL Internalizing problems (**B**) and BRIEF Working memory (**C**) scale ratings (y-axis) with statistics for the Pearson correlation overlaid.

Based on prior work showing that amygdala rsfMRI connectivity to the ventromedial prefrontal cortex contributes to internalizing problems (45–47), we tested whether rFC in the ventrolateral amygdala network (Figure 4A) predicted internalizing ratings in the TBI group (N = 16). Indeed, patients with the greatest ventrolateral amygdala connectivity were rated by their parents to have the most internalizing problems (Figure 4B). This finding could not be explained by potential confounds, including age, sex, handedness, time since injury, severity of injury (GCS on arrival), or presence of gross injuries on hospital CT (as described in Table 1), uncinate fasciculus FA, ventrolateral amygdala MD, or amygdala volume, in that these variables did not correlate with both amygdala connectivity and the behavioral variables. Further, supporting the specificity of this brain-behavior relationship to the affective domain, we found no significant correlation between ventrolateral amygdala connectivity and ratings on the Working Memory subscale of the BRIEF (Figure 4C).

### Internalizing problems: voxelwise analysis

**Figure 5:**
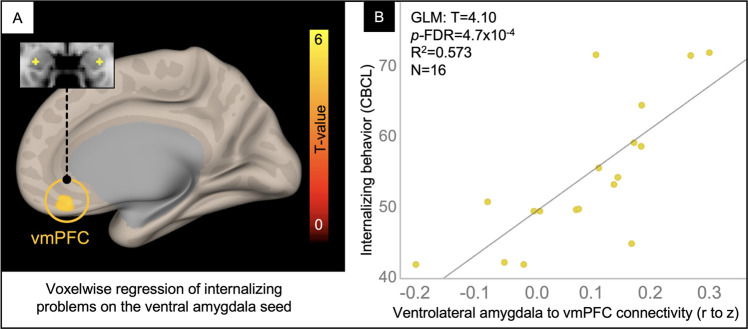
Voxelwise correlation between CBCL Internalizing Problem ratings and ventrolateral amygdala connectivity. Resultant clusters of voxels in the ventromedial prefrontal cortex (vmPFC) for the regression of Internalizing problem ratings from the CBCL on ventrolateral amygdala rFC (**A**, *p*-uncorrected < 0.01 with cluster-size *p*-FDR corrected < 0.05). Scatter plot (**B**) showing the correlation between rFC of the ventrolateral amygdala to peak voxel in the vmPFC cluster (x-axis) and Internalizing Problem ratings from the CBCL (y-axis) with overlaid statistics for the GLM (given these were derived from the voxelwise regression).

In a voxelwise analysis of the ventrolateral amygdala network, patients in the TBI group with the highest internalizing problem ratings had the greatest connectivity between the ventrolateral amygdala and voxels in the ventromedial prefrontal cortex (vmPFC), explaining 57% of the variance in internalizing problems (Figure 5).

### Healthy control group comparison

Supporting the clinical significance of these brain-behavior correlations, all behavioral ratings were higher (less well-controlled) in the TBI group as compared to the HC group (Emotional Control: *T *= 2.26, *p = *0.028; Externalizing: *T *= 2.34, *p *= 0.02), although not Internalizing (*T *= 0.36, *p *= 0.71). The reported brain-behavior associations were specific to the TBI population in that healthy controls did not show a correlation in amygdala connectivity with emotional control, externalizing or internalizing problems (Pearson correlation *p-*values ranged from 0.78–0.93). In addition, medial and ventrolateral amygdala connectivity with the peak voxels from the voxelwise analyses did not differ across groups (Independent *t*-test *p-*values ranged from 0.11–0.35).

### Discussion

This study is unique in that it combined functional connectivity from rsfMRI with measures of macro- and microstructure to investigate the mechanisms of mood and behavioral dysregulation after msTBI in adolescents. Findings from this study suggest that specific functional circuits within the amygdala's broad connectome, particularly its frontoamygdala circuitry, explain significant variance in affective and behavioral problems that are hallmarks of chronic TBI. We showed that the functional connectivity predictors of externalizing and internalizing problems were differentiable at the amygdala subregional level and could not be better explained by injury severity, gross lesion burden, time since injury, patient demographics, or structural measures of amygdala circuit integrity, diffusivity, and volume.

### Externalizing problems

For adolescents in the chronic phase after msTBI, medial amygdala network rFC predicted problems in emotional control and externalizing behavior that were not accounted for by measures of structural aberrancy or injury severity. Medial amygdala connectivity to voxels in the rACC and PCC drove this relationship, predicting upwards of 48% of the variance in externalizing problems (Figure 3). This link was specific to medial amygdala connectivity to these structures rather than their connectivity with each other. The brain-behavior association was also specific to affective rather than cognitive problems.

The regions in the medial amygdala network play a critical role in integrating memories and goals with salient information from the environment, particularly the salient actions of others, to regulate affective, approach, and affiliative behaviors (39). In prior work, we found that atrophy specific to the medial amygdala network, over and above atrophy to other amygdala networks, predicts decreased empathy and warmth as well as increased disregard and sometimes cold or cruel behavior towards other people in a sample of patients with frontotemporal dementia (41).

Consistent with this role and the findings from the current study, prior work in typically developing adolescents found that elevated rFC between the amygdala and rACC predicted greater externalizing behaviors (42) Connectivity between these structures also increases through development and correlates with increases in externalizing behaviors, a finding that was unique to the amygdala's connectivity to the rostral portion of the ACC (42) The positive direction of this brain-behavior association extends to a sample of adolescents with conduct disorder and callous-unemotional traits who had increased rFC between the amygdala and a cluster extending from the rACC to the PCC as compared to typically developing controls, and increased connectivity between the amygdala and rACC correlated with higher callous-unemotional traits in the adolescents with conduct disorder (43). Additional work in adolescents with conduct disorder also links heightened amygdala rFC with rACC and PCC to greater degrees of aggression (44). These prior findings suggest heightened rFC between the amygdala and portions of the cingulate cortex may result in weaker regulatory control over externalizing behaviors, such as aggressiveness, disinhibition, or a disregard for other people.

### Internalizing problems

For adolescents in the chronic phase after msTBI, ventrolateral amygdala network rFC (yellow network in Figure 1), particularly connectivity to voxels in the ventromedial prefrontal cortex (vmPFC), explained 57% of the variance in internalizing problems (Figure 5). This is consistent with a meta-analysis of studies using rsfMRI of the amygdala to predict internalizing problems, which showed that there were two distinct anterior cingulate clusters related to mood dysregulation, the more ventral cluster specific to studies of at-risk youth who had been exposed to aversive childhood events (48). Considering this prior work alongside our findings suggests that heightened rFC in frontoamygdala pathways may translate to mood dysregulation or a decreased ability to downregulate negative moods, such as anxiety, in adolescents after msTBI.

Pre-clinical work demonstrates parallel and more causative insights into the mechanisms linking connectional differences after injury with internalizing problems. Specifically, prior studies have shown cellular structural differences with increased dendritic arborization in the basolateral amygdala (59) as well as increased excitatory proteins after TBI that was associated with increased fear learning (60). Another study found increased activation in the auditory thalamo-amygala pathway during noxious auditory stimuli that was associated with heightened sensitivity and defensive behavior to the stimuli in rodents after moderate TBI but not in uninjured sham animals (61). This suggests a hyperconnected and overactive arousal circuit due to TBI and fits well with the functional and structural anatomy predicting internalizing symptoms in our human cohort.

### Limitations

Our study had many limitations in common with other studies in TBI but also unique to this specific cohort. In general, studies of patients with TBI suffer from a heterogeneity in injury characteristics, including different severities, mechanisms, intracranial lesions, neuropsychological history, and more. We attempted to control for many of these and other obvious demographic confounds as well as measures of macro- and microstructure that could account for the observed findings in rsfMRI. None explained our main findings. While the sample size is relatively comparable to other similar studies of this population in the literature, it is quite small, and does not allow subgroup analyses by sex, age, severity, mechanism, or other potentially confounding variables. In future work, larger samples will enable investigation of more subtle effects of potential confounds, particularly sex given its role in amygdala development and function. These findings will clearly need replication as recent work demonstrates poor replicability in brain-wide association studies involving less than thousands of subjects (62). Further discussion of this replicability problem suggests that a practical alternative to such large samples is to perform a focused study in which the phenotype of interest is deeply characterized (63). We believe one of the strengths in the present cohort is the detailed, and hypothesis driven, behavioral and imaging data that we used to define the behavioral and neural phenotype of interest while ruling out other, closely related potential confounds. Altogether, at this point, rsfMRI does not seem to be a usable measure on the individual subject level, but it does continue to explain variance in behavior between people and cohorts at the group level and may serve to guide brain-specific treatments, such as forms of neuromodulation.

## Conclusions and future directions

Whereas markers of injury severity, including GCS and pathological findings on CT, did not explain differences in affective and behavioral outcomes in our adolescents with msTBI, aberrancies in the functional connectome of limbic circuitry did account for problems in externalizing and internalizing behavior. The results were specific to circuits situated in the limbic components of the medial frontal and temporal lobes rather than other limbic or more neocortical circuits. This could reflect a specific process of recovery, compensation, or premorbid vulnerability in the TBI group that reflects recruitment of ongoing reparative processes, additional effort needed, or chronic alteration, damage, among other possibilities. Current treatments do not target the underlying etiology of affective disability in pediatric TBI. Rather, the majority target the specific symptoms of traumatic brain injury, such as antidepressants for mood problems, cognitive-behavioral therapy for anxiety, or family therapy for academic social adjustment (64). This circuitry serves as an excellent potential target for TBI therapy given that it contains many of the regions vulnerable to traumatic brain injury, underlies normal and pathologic variations in affective behavior (38, 39, 41), and undergoes protracted development in youth (65). Future work will focus on neuromodulation techniques to directly affect frontoamygdala circuits with the aim to mitigate affect and behavior regulation problems after TBI.

## Data Availability

The raw data supporting the conclusions of this article will be made available by the authors, without undue reservation.
